# Influence of Urbanicity and County Characteristics on the Association between Ozone and Asthma Emergency Department Visits in North Carolina

**DOI:** 10.1289/ehp.1306940

**Published:** 2014-02-25

**Authors:** Jason D. Sacks, Ana G. Rappold, J. Allen Davis, David B. Richardson, Anna E. Waller, Thomas J. Luben

**Affiliations:** 1 National Center for Environmental Assessment, Office of Research and Development, and; 2 National Health and Environmental Effects Research Laboratory, U.S. Environmental Protection Agency, Research Triangle Park, North Carolina, USA; 3 Department of Epidemiology, and; 4 Department of Emergency Medicine, University of North Carolina at Chapel Hill, Chapel Hill, North Carolina, USA

## Abstract

Background: Air pollution epidemiologic studies, often conducted in large metropolitan areas because of proximity to regulatory monitors, are limited in their ability to examine potential associations between air pollution exposures and health effects in rural locations.

Methods: Using a time-stratified case-crossover framework, we examined associations between asthma emergency department (ED) visits in North Carolina (2006–2008), collected by a surveillance system, and short-term ozone (O_3_) exposures using predicted concentrations from the Community Multiscale Air Quality (CMAQ) model. We estimated associations by county groupings based on four urbanicity classifications (representative of county size and urban proximity) and county health.

Results: O_3_ was associated with asthma ED visits in all-year and warm season (April–October) analyses [odds ratio (OR) = 1.019; 95% CI: 0.998, 1.040; OR = 1.020; 95% CI: 0.997, 1.044, respectively, for a 20-ppb increase in lag 0–2 days O_3_]. The association was strongest in Less Urbanized counties, with no evidence of a positive association in Rural counties. Associations were similar when adjusted for fine particulate matter in copollutant models. Associations were stronger for children (5–17 years of age) compared with other age groups, and for individuals living in counties identified with poorer health status compared with counties that had the highest health rankings, although estimated associations for these subgroups had larger uncertainty.

Conclusions: Associations between short-term O_3_ exposures and asthma ED visits differed by overall county health and urbanicity, with stronger associations in Less Urbanized counties, and no positive association in Rural counties. Results also suggest that children are at increased risk of O_3_-related respiratory effects.

Citation: Sacks JD, Rappold AG, Davis JA Jr, Richardson DB, Waller AE, Luben TJ. 2014. Influence of urbanicity and county characteristics on the association between ozone and asthma emergency department visits in North Carolina. Environ Health Perspect 122:506–512; http://dx.doi.org/10.1289/ehp.1306940

## Introduction

Experimental and epidemiologic studies have consistently demonstrated respiratory effects due to short-term ozone (O_3_) exposures [U.S. Environmental Protection Agency (EPA) 2013a]. As part of this evidence, multiple epidemiologic studies have reported positive and statistically significant associations between short-term ambient O_3_ exposures and asthma emergency department (ED) visits (Stieb et al. 2009; Strickland et al. 2010), especially during the warm or summer season when O_3_ concentrations are higher (Ito et al. 2007; Strickland et al. 2010).

The single- and multi-city epidemiologic studies providing the scientific evidence indicating associations between air pollution and health outcomes, including short-term O_3_ exposures and asthma ED visits, generally include studies conducted in large metropolitan areas that rely on air quality data from regulatory networks. As a result, these studies are unable to answer a key question that often arises during the review of the National Ambient Air Quality Standards (NAAQS): Are the associations observed in large metropolitan areas consistent with those observed in less urban and rural locations?

Recently, investigators have begun to develop statistical approaches and models that allow for the spatial and temporal characterization of air quality in areas of the country, including rural locations, where monitoring data are limited or unavailable (Bell 2006). One such model, developed by the U.S. Environmental Protection Agency (EPA)—the Community Multiscale Air Quality (CMAQ) model (U.S. EPA 2013b)—predicts air pollutant concentrations for the entire United States to defined grid cells (12 km × 12 km in the eastern United States; 36 km × 36 km in the western United States) by bringing together information from meteorological, emissions, and air chemistry-transport models. To develop more refined air pollutant predictions, a hierarchical Bayesian space–time model was developed that fuses monitored data with the 12-km gridded output from CMAQ to predict average concentrations for individual CMAQ grid cells (McMillan et al. 2010). This model was further refined using a downscaler approach to predict air quality concentrations to a specific point (e.g., U.S. Census tract) (Berrocal et al. 2010a, 2010b, 2012).

North Carolina has in place a statewide surveillance system, the North Carolina Disease Event Tracking and Epidemiologic Collection Tool (NC DETECT), which provides data on ED visits for the 116 of 120 hospitals in the state with EDs operating 24 hr/day. Using these data we examined the association between estimates of short-term O_3_ exposures and asthma ED visits in North Carolina for the years 2006–2008. To evaluate differences in the risk of O_3_-related asthma ED visits in counties that have different levels of urbanization, counties were categorized by degree of urbanicity. In addition, the influence of overall county health on the association between short-term O_3_ exposures and asthma ED visits was assessed using the 2012 County Health Rankings for North Carolina.

## Methods

*Asthma ED visit data*. Daily asthma ED visits for North Carolina for the period of 1 January 2006 through 31 December 2008 were obtained from NC DETECT (2010). Using the *International Classification of Diseases, 1975 Revision* (ICD-9; World Health Organization 1977), we defined asthma ED visits as those with a primary ICD-9 diagnosis code of 493.0 to 493.9, for all ages. Each case was classified according to their county of residence at the time of ED visit.

NC DETECT was established in 2004 through the North Carolina Division of Public Health and the University of North Carolina at Chapel Hill School of Medicine, Department of Emergency Medicine. Starting in 2005, a statewide mandate began requiring all acute care hospital-affiliated EDs in North Carolina to submit data on ED visits to the state. Of the 113 EDs covered by the mandate, 64 were reporting to NC DETECT at the start of the year 2006, and 88 at the end of the year. In 2007 and 2008, NC DETECT was capturing an estimated 92.5% and 99%, respectively, of all ED visits in the state (A.E.W., personal communication). The Human Subjects Institutional Review Board of the University of North Carolina at Chapel Hill and the Environmental Protection Agency approved the present study.

*Air pollution and meteorological data*. Daily maximum 8-hr average O_3_ and daily 24-hr average PM_2.5_ [particulate matter (PM) mass with an aerodynamic diameter ≤ 2.5 μm) CMAQ model estimates for the years 2006 to 2008 predicted to the centroid of each U.S. Census tract (from the 2000 Census) in North Carolina using the Bayesian space–time downscaler approach were obtained from the U.S. EPA (2012a). Predictions were not available for 31 December for the year 2006 because complete daily CMAQ output was not available due to conversion from Greenwich Mean Time to local Eastern Time (U.S. EPA 2012b).

Mean temperature and mean dew point temperature data for each county in North Carolina were obtained from the State Climate Office of North Carolina (North Carolina State University 2010). Of the 100 counties in North Carolina, 94 counties had temperature data, and 75 had both temperature and dew point temperature data. To obtain complete weather data for all 100 counties, the missing mean temperature and mean dew point temperature data were imputed by taking the average of the mean temperature and/or mean dew point temperature of all counties that bordered the county that was missing data.

*Exposure assignment*. The CMAQ model using the Bayesian space–time downscaler approach predicted O_3_ and PM_2.5_ concentrations to the year 2000 U.S. Census tract centroids. Data obtained from NC DETECT (2010) provided the county of patient residence for each asthma ED visit. Thus, the spatial resolution of the predicted O_3_ and PM_2.5_ concentrations obtained from CMAQ and the asthma ED visit data were different. Therefore, predicted O_3_ and PM_2.5_ concentrations were aggregated by performing an area-weighted average of U.S. Census tract centroids to the county level so that exposure data would be at the same spatial resolution as the health outcome data.

*Statistical analysis*. The association between short-term exposures to air pollution and asthma ED visits was examined using a time-stratified case-crossover approach. Referent days were selected as the same day of the week within the same month and year as the case day (i.e., ED visit). Therefore, for each case, depending on the month, 3 or 4 referent days were selected. Individuals that entered the ED more than once in a month were not excluded from the analysis because it was not possible to identify individuals with a repeat visit due to the construct of the data (i.e., deidentified).

To control for the potential confounding effects of weather, a model was constructed that included covariates for same-day (lag 0) mean temperature and mean dew point temperature with natural splines and 4 degrees of freedom (df). This model was selected based on an initial analysis of statistical models, each of which used a different approach to control for the potential confounding effects of weather, from studies that examined associations between short-term air pollution exposures and asthma or respiratory-related ED visits (Lavigne et al. 2012; Villeneueve et al. 2007; Zanobetti and Schwartz 2006). We found that each of these models yielded comparable results (i.e., the difference in risk estimates across models was primarily < 1%, with the largest difference being < 10%). As a result, the model used in the present study is based on Lavigne et al. (2012) because their model controls for potential nonlinear effects of both temperature and dew point temperature.

Associations between short-term O_3_ exposure and asthma ED visits were examined in both all-year and warm season (April–October) analyses using a conditional logistic regression model in R, version 2.15.0, statistical software (R Foundation for Statistical Computing, Vienna, Austria). Analyses were limited to examining associations using the average of O_3_ concentrations on the day of the ED visit or reference day, and the 2 days prior to the ED visit or reference day (lag 0–2), based on previous studies demonstrating the strongest association between short-term O_3_ exposures and asthma ED visits within the first few days after exposure (Ito et al. 2007; Stieb et al. 2009; Strickland et al. 2010; Villeneuve et al. 2007). Odds ratios (ORs) were estimated for a 20-ppb increase in maximum 8-hr average O_3_ concentrations on ED visit days compared with reference days, representing an approximate interquartile range increase in O_3_ concentrations across North Carolina.

*Copollutant confounding*. Currently the downscaler CMAQ model only predicts O_3_ and PM_2.5_ concentrations. As such, the potential confounding effect of PM_2.5_ on the O_3_–asthma ED visits relationship was examined in copollutant models using lag 0–2 days PM_2.5_ concentrations. In addition, the O_3_–asthma ED visits relationship was examined by stratifying on days where PM_2.5_ concentrations were considered high and relatively low (i.e., > 75th percentile of the PM_2.5_ distribution and < 50th percentile of the PM_2.5_ distribution, respectively).

*Urbanicity classifications*. Counties were classified into four urbanicity categories based on the U.S. Department of Agriculture’s (USDA) Rural-Urban Continuum Codes (RUCCs) (USDA 2012). The RUCC classification scheme “distinguishes metropolitan counties by size and nonmetropolitan counties by degree of urbanization and proximity to metro areas” (USDA 2012). Counties were classified into the following four categories: *a*) Metro Urban counties (counties in metro areas with a population ranging from ≤ 250,000 to ≥ 1 million), *b*) Non-Metro Urban counties (urban population ≥ 20,000), *c*) Less Urbanized counties (urban population ranging from 2,500 to < 20,000), and *d*) Rural counties (completely rural or urban population ≤ 2,500), as detailed by Luben et al. (2009).

*Effect measure modification*. Two approaches were used to examine potential factors that could modify the risk of O_3_-related asthma ED visits. In the first approach, the 2012 County Health Rankings for North Carolina for health outcomes (i.e., mortality, morbidity) and health factors (i.e., health behaviors, clinical care, social/economic factors) were used to examine whether the relative health of each county would modify the association between O_3_ exposures and asthma ED visits (County Health Rankings and Roadmaps 2012). For this analysis, based on the County Health Rankings for North Carolina, the 100 counties were classified into quartiles for each health outcome and health factor. Counties in the first quartile represented those counties with a better health ranking. Effect measure modification of the O_3_–asthma ED visit association was then examined by quartiles for each health outcome and health factor.

In the second approach, to examine potential factors that could modify the risk of O_3_-related asthma ED visits, the statewide results were stratified by age and sex. The age-stratified analysis focused on four age groups: < 5, 5–17, ≥ 18, and ≥ 65 years of age.

## Results

For the years 2006–2008, a total of 122,607 North Carolina ED visits with a primary diagnosis of asthma were reported to NC DETECT (2010). Of these asthma ED visits (cases), 986 were excluded from this analysis because we were unable to calculate the lag 0–2 day exposure metric for ED visits on days prior to 3 January 2006. In addition, case days and referent days that relied on data for 31 December 2006 were excluded because CMAQ predictions did not include output for 31 December due to the conversion from Greenwich Mean Time to local Eastern Time (U.S. EPA 2012b). After the exclusion of cases and referents with incomplete exposure data, 121,621 asthma ED visits and 532,079 total observations (i.e., case days and referent days) remained (Table 1). Overall, the number of asthma ED visits varied across urbanicity classifications, which is consistent with the population distribution of the state (State of North Carolina 2011), with 70.5% of asthma ED visits occurring for patients living in Metro Urban, 19.5% in Non-Metro Urban, 7.7% in Less Urbanized, and 2.4% in Rural counties. The distribution of cases by county urbanicity classification was similar across individual years and when limited to the warm season.

**Table 1:** Asthma ED visits and air pollution concentrations for North Carolina, statewide and by urbanicity classification, 2006–2008.

Category	Counties (*n*)	Cases (*n*)	Observations (*n*)	O_3 _(ppb)			PM_2.5_ (μg/m^3^)		
				Mean	75th percentile	Maximum	Mean	75th percentile	Maximum
All-year
Statewide	100	121,621	532,079	43.6	54.3	108.1	12.2	15.5	94.1
Metro Urban	40	85,724	375,110	43.5	54.5	108.1	12.3	15.7	84.1
Non-Metro Urban	19	23,682	103,516	43.7	54.2	92.7	12.2	15.5	62.5
Less Urbanized	20	9,301	40,688	43.9	53.3	94.6	11.5	14.5	57.5
Rural	21	2,914	12,765	44.8	53.9	87.0	10.8	13.6	94.1
Warm season
Statewide	100	69,287	305,919	50.1	59.2	108.1	13.2	16.8	94.1
Metro Urban	40	48,717	215,141	50.3	59.7	108.1	13.4	17.0	84.1
Non-Metro Urban	19	13,524	59,646	49.8	58.8	92.7	13.1	16.6	62.5
Less Urbanized	20	5,351	23,643	49.0	57.4	94.6	12.3	15.6	57.5
Rural	21	1,695	7,489	49.5	57.5	87.0	11.7	15.0	94.1

Of the total number of asthma ED visits during the study period, the majority were in females, for both the entire state and in each urbanicity classification (Table 2). The age distribution of asthma ED visits was similar by urbanicity classification, 0–4 years (12.2–14.4%), 5–17 years (22.8–24.7%), ≥ 18 years (60.8–64.7%), and ≥ 65 years (5.7–9.9%). Across the years included in the analysis, the number of asthma ED visits was similar in 2007 and 2008; the inclusion of more hospitals reporting to NC DETECT in 2007 and 2008 resulted in the number of asthma ED visits increasing by 28–37% statewide, as well as in Metro Urban, Non-Metro Urban, and Less Urbanized counties, relative to 2006. However, in the Rural counties, the number of asthma ED visits increased from 620 in 2006 to over 1,000 in both 2007 and 2008 (NC DETECT 2010), an approximate 40% increase (Table 2).

**Table 2:** Number (%) of asthma ED visits by sex, age, and year, statewide and by urbanicity classification, North Carolina, 2006–2008.

Characteristic	Statewide	Metro Urban	Non-Metro Urban	Less Urbanized	Rural
Total no. of cases in analyses	121,621	85,724	23,682	9,301	2,914
Sex
Males	53,923 (44.3)	38,407 (44.8)	10,133 (42.8)	4,100 (44.1)	1,283 (44.0)
Females	67,698 (55.7)	47,317 (55.2)	13,549 (57.2)	5,201 (55.9)	1,631 (56.0)
Age group (years)
0–4	16,860 (13.9)	12,367 (14.4)	2,956 (12.5)	1,137 (12.2)	400 (13.7)
5–17	29,434 (24.2)	21,210 (24.7)	5,396 (22.8)	2,200 (23.7)	628 (21.6)
≥ 18	75,327 (61.9)	52,147 (60.8)	15,330 (64.7)	5,964 (64.1)	1,886 (64.7)
≥ 65	7,747 (6.3)	4,928 (5.7)	1,815 (7.7)	716 (7.7)	288 (9.9)
Year
2006	31,340 (25.8)	22,456 (26.2)	5,962 (25.2)	2,302 (24.8)	620 (21.3)
2007	43,997 (36.2)	30,827 (36.0)	8,734 (36.9)	3,354 (36.1)	1,082 (37.1)
2008	46,284 (38.1)	32,441 (37.8)	8,986 (37.9)	3,645 (39.2)	1,212 (41.6)

Mean maximum 8-hr average O_3_ concentrations across all years were similar when stratified by urbanicity classification, ranging from 43.5–44.8 ppb (Table 1). The extent of temporal correlation in O_3_ concentrations within urbanicity classifications was found to be high, with correlation coefficients (*r*) ranging from 0.97 to 0.99. Across urbanicity classifications, 24-hr average PM_2.5_ concentrations decreased as counties became Less Urbanized. The high maximum PM_2.5_ concentrations observed across all four urbanicity classifications can be attributed to a peat bog wildfire in June 2008 that affected a large portion of the state (Rappold et al. 2011). Statewide, O_3_ and PM_2.5_ were moderately correlated (*r* = 0.54). In the warm season when compared with all-year, mean maximum 8-hr average O_3_ concentrations were higher by 4.7–6.8 ppb across urbanicity classifications, with PM_2.5_ concentrations uniformly higher by approximately 1 μg/m^3^.

**Figure 1:**
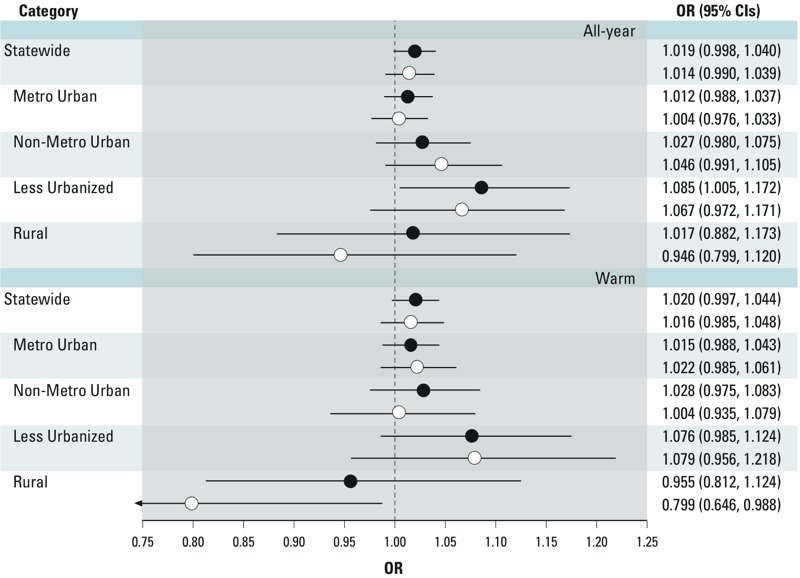
Association between short-term (lag 0–2 days) O_3_ (95% CI) exposures and asthma ED visits in single and copollutant models with PM_2.5_ for all-year and warm season analyses for North Carolina for a 20-ppb increase in maximum 8-hr average O_3_ concentrations, statewide and by urbanicity classification, 2006–2008. Regression models adjusted for same-day mean temperature and mean dew point temperature using natural splines and 4 df. Solid circles represent single pollutant models with O_3_; open circles represent copollutant models adjusted for PM_2.5_.

In the statewide analysis, the association between short-term O_3_ exposures and asthma ED visits was similar in magnitude for both all-year (OR = 1.019; 95% CI: 0.998, 1.040) and warm season (OR = 1.020; 95% CI: 0.997, 1.044) analyses (Figure 1). In both the all-year and warm season analyses, the association of largest magnitude was observed for the Less Urbanized counties with an OR = 1.085 (95% CI: 1.005, 1.172) for all-year and OR = 1.076 (95% CI: 0.985, 1.124) for the warm season. The association between O_3_ and asthma ED visits in Rural counties was small in magnitude with larger uncertainty in the all-year analysis and null in the warm season analysis. In analyses of associations between asthma ED visits and PM_2.5_, the magnitude of associations with asthma ED visits was similar to O_3_ in the statewide analysis and for the Metro Urban classification; however, the associations were smaller for the Non-Metro Urban and Rural classifications (results not presented). In copollutant models with PM_2.5_, estimates of O_3_-asthma ED visit associations by urbanicity classification were of similar magnitude, but lower precision, in the statewide analysis. There was attenuation of effect estimates in the Non-Metro Urban and Rural county classifications (Figure 1). To further examine the potential confounding effects of PM_2.5_ on the relationship between short-term O_3_ exposures and asthma ED visits, asthma ED visit cases were stratified into days with high (i.e., PM_2.5_ concentrations > 75th percentile of the PM_2.5_ distribution) and low (i.e., PM_2.5_ concentrations < 50th percentile of the PM_2.5_ distribution) PM_2.5_ concentrations. A total of 90,337 cases were classified into one of these categories (27,856 > 75th percentile and 62,481 < 50th percentile). In an all-year analysis, the magnitude of the O_3_ effect was larger when stratifying on days < 50th percentile compared with > 75th percentile (Table 3).

**Table 3:** Association (95% CI) between short-term (lag 0–2 days) O_3_ exposures and asthma ED visits by potential effect measure modifiers for North Carolina for a 20-ppb increase in maximum 8-hr average O_3_ concentrations, statewide, 2006–2008.

Effect measure modifier	OR (95% CI)
Sex
Male	1.027 (0.996, 1.058)
Female	1.013 (0.985, 1.041)
Age (years)
< 5	0.996 (0.943, 1.053)
5–17	1.079 (1.035, 1.125)
≥ 18	1.001 (0.975, 1.027)
≥ 65	0.996 (0.917, 1.082)
PM concentrations
< 50th percentile (11.1 μg/m3)	1.017 (0.983, 1.051)
> 75th percentile (15.5 μg/m3)	1.002 (0.946, 1.060)
Regression models adjusted for same-day mean temperature and mean dew point temperature using natural splines and 4 df.	

In addition to examining whether associations between O_3_ and asthma ED visits differed according to urbanicity classification, we also examined potential effect measure modification by demographic characteristics and County Health Rankings (Table 3). There was a modest indication of a stronger association in males than females. The association appeared to be limited to children 5–17 years of age (OR 1.079; 95% CI: 1.035, 1.125), with no evidence of associations in other age groups (< 5, ≥ 18, or ≥ 65 years of age) (Table 3).

**Figure 2:**
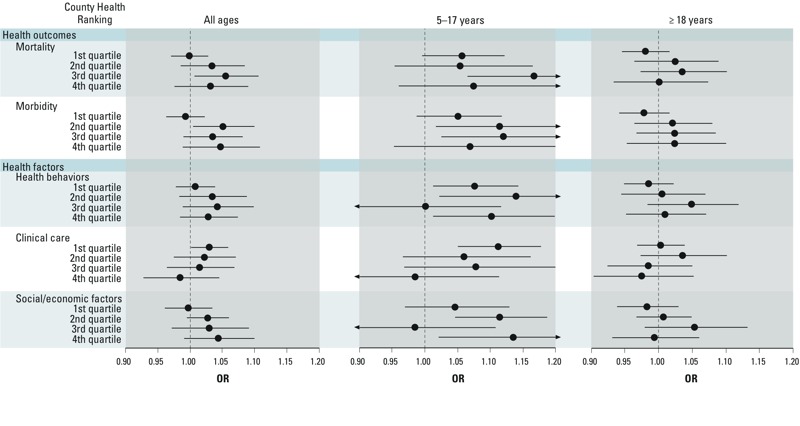
Association between short-term (lag 0–2 days) O_3_ exposures and asthma ED visits for North Carolina by County Health Rankings for health outcomes and health factors by age—All-Year, 2006–2008. For the County Health Rankings, the first quartile represents counties with better health rankings, whereas the fourth quartile represents counties with poorer health rankings. ORs (95% CI) were calculated for a 20-ppb increase in maximum 8-hr average O_3_ concentrations. Regression models were adjusted for same-day mean temperature and mean dew point temperature using natural splines and 4 df.

When examining associations by County Health Ranking for all ages, across the health outcomes and health factors, except clinical care, no association was observed between O_3_ concentrations and asthma ED visits among those living in counties in the first County Health Ranking quartile, whereas ORs were > 1 for counties with lower health rankings (Figure 2). Patterns of associations according to County Health Ranking quartiles differed when stratified by age (i.e., 5–17 or ≥ 18 years of age), although differences must be interpreted with caution given the larger uncertainty in quartile-specific ORs, particularly for the younger age group. Among children, associations were positive across all quartiles for the health outcomes; whereas for adults, the pattern was similar to the all-ages analysis. For the health behaviors and social and economic health factors, associations between O_3_ and asthma ED visits were positive except among children living in counties in the third quartiles for each factor, which is the inverse of what was observed for adults. The pattern of associations across quartiles for children for the clinical care health factor was similar to the all-age analysis, whereas for adults, a positive association was only observed for the second quartile.

## Discussion

Epidemiologic studies of daily changes in air pollution have primarily been limited to large metropolitan cities because of their reliance on data from regulatory monitoring networks. The CMAQ model provides an additional resource for exposure estimation to examine the public health impact of air pollution, specifically in locations not previously examined because of the lack of air quality data. In the present study, we demonstrate the utility of the CMAQ model in epidemiologic studies by examining short-term O_3_ exposures among counties with varying levels of urbanicity and measures of overall county health. The results of the present North Carolina study suggest that associations between short-term O_3_ exposures and asthma ED visits are not limited to large metropolitan areas. In addition, adjusting for PM_2.5_ concentrations had minimal influence on associations, although adjusted estimates showed greater attenuation in some subgroups when stratified by season and urbanicity. the present study also suggests that the O_3_-asthma ED visit association may be influenced by overall county health as well as the age of the population.

In North Carolina, the magnitude of the association between short-term O_3_ exposures and asthma ED visits was similar (i.e., within approximately 5%) in all-year and warm season analyses. However, compared with studies conducted by Lavigne et al. (2012) and Stieb et al. (2009), which examined the association between short-term O_3_ exposures and asthma ED visits using 1-hr maximum and 24-hr average exposure metrics, but relatively similar increments (18.4 and 26 ppb, respectively), the all-year results for this analysis are smaller in magnitude. The all-year results were also smaller in magnitude compared with a study conducted in New York City by Ito et al. (2007), which examined the association between short-term O_3_ exposures and asthma ED visits using the maximum 8-hr average exposure metric. Although Ito et al. (2007) used an increment of approximately 60 ppb, when standardizing the results of both Ito et al. (2007) and our analysis to a 30-ppb increase in maximum 8-hr average concentrations [which is representative of a low-to-high change in O_3_ concentrations (i.e., annual mean to 95th percentile difference in maximum 8-hr average concentrations in the United States; U.S. EPA 2013a)], the all-year estimate for North Carolina was still approximately 48% smaller. The associations observed in the present study were smaller in magnitude even though mean 8-hr maximum O_3_ concentrations in North Carolina were higher across all years (30.4 vs. 43.6 ppb) and in the warm season (42.7 vs. 50.1 ppb) compared with New York City. The difference in the magnitude of associations could be attributed to the aforementioned studies being conducted in one or more large metropolitan areas, whereas the present study encompassed the entire state of North Carolina and, subsequently, locations with less urbanization. In addition, it is important to note that the difference in the magnitude of associations between studies for the all-year and warm season analyses could be due to the overall difference in seasons (e.g., season length, daily temperature) between study locations.

Interestingly, in analyses of urbanicity the magnitude of the association was largest in the Less Urbanized counties, with no association with asthma ED visits observed in Rural counties. These results suggest that simply generalizing the associations reported in epidemiologic studies that focus on only large metropolitan areas to Less Urbanized or Rural locations may be misleading. It is possible that exposure misclassification is greater in Rural counties, which could introduce bias that would likely be toward the null, and may contribute to, or explain, the essentially null association in Rural counties. Exposure misclassification by urbanicity could also be due to differences in neighborhood-scale variability in O_3_ concentrations, specifically because of nitrogen oxide (NO) scavenging. In this case, when there is an abundance of NO, such as in areas with heavy traffic, it can lead to lower O_3_ concentrations (Roberts-Semple et al. 2012). Another source of potential exposure misclassification could result if there is a varied level of agreement in downscaler CMAQ O_3_ predictions and ambient O_3_ concentrations by level of urbanicity. Although this is difficult to assess because of the relative sparsity of monitors in rural areas, it has been demonstrated that CMAQ does contain information that is useful for predicting O_3_ concentrations at unmonitored sites (Berrocal et al. 2012).

Another possible explanation for the differences observed is that residents of Rural counties may lack access to hospital EDs, and thus the way they respond behaviorally to asthma exacerbations, such as driving to the nearest hospital, could differ from residents of more urban counties. In addition, regular access to a physician may vary by urbanicity, leading to different levels of asthma management and subsequently different rates of asthma ED visits. However, the NC DETECT database does not include individual-level data that would provide details such as accessibility to a hospital, distance to nearest hospital from residence, or asthma management status for each individual case. It is also important to note that our estimates of association had larger uncertainty when stratified by urbanicity, and differences among groups should therefore be interpreted with caution.

Within the present study there was some indication that males may be at increased risk of asthma ED visits in response to O_3_ exposure. However, studies of asthma ED visits, as well as respiratory-related hospital admissions, have demonstrated limited evidence of differences in associations by sex (U.S. EPA 2013a). When examining differences in associations by age, studies have consistently demonstrated that children are at increased risk of asthma hospital admissions and ED visits (e.g., Strickland et al. 2010; U.S. EPA 2013a; Villeneuve et al. 2007), which is further supported by the stronger association between O_3_ concentrations and asthma ED visits in children 5–17 years of age in our study population. Short-term O_3_ exposures were not associated with asthma ED visits among children < 5 years of age, but this finding should be interpreted with caution given the difficulty in diagnosing asthma in young children, who often experience transient wheeze (National Heart Lung and Blood Institute 2007).

The results of an examination of County Health Rankings found evidence that the association between short-term O_3_ exposures and asthma ED visits across all ages was larger in magnitude in counties with a lower health ranking for both health outcomes, and the health behaviors and social and economic health factors. The pattern of associations for each health outcomes (i.e., mortality and morbidity, which encompass years of potential life lost before 75 years of age and measures of quality of life and poor birth outcomes, respectively) add support to the findings that individuals with preexisting conditions may be at-risk of O_3_-related health effects because both of these outcomes are measuring the underlying health of each county (U.S. EPA 2013a).

A closer examination of the health behaviors and social and economic health factors finds that they are interrelated and consist of components that are often used in epidemiologic studies to examine the influence of socioeconomic status on the association between air pollution exposures and health effects. Together, these health factors suggest that those counties with a larger percentage of the population with poorer health behaviors and socioeconomic status may be at-risk of an asthma ED visit in response to short-term O_3_ exposures. These results add to the growing body of evidence suggesting that socioeconomic status is an important factor to consider when characterizing those populations potentially at-risk of an air pollution–related health effect (Rappold et al. 2012; U.S. EPA 2013a).

Of the health factors examined in the all-age analysis, the clinical care factor was the only factor where associations were larger in magnitude in the counties with better access and quality of health care. This pattern of associations was also observed in the analysis focusing on children, but the associations had larger uncertainty. The larger asthma ED visits associations in these counties could be an artifact of access to health care and subsequently having a larger percentage of the population with health insurance compared with the next lowest quartile as well as with counties having a worse health ranking having less access to health care.

Although the results of the present study are consistent with those reported in epidemiologic studies that use more traditional methods for exposure assessment (i.e., air quality measurements obtained from central site monitors), there are benefits and disadvantages to using the CMAQ model with the downscaler approach to predict air pollutant concentrations. The ability to predict air pollutant concentrations for a large spatial extent allows for the use of health data for an entire state instead of being limited to large metropolitan areas. This ultimately allows for a better characterization of the public health impact of air pollution exposures in areas of the country underrepresented in the current epidemiologic literature. In addition, the downscaler approach is able to better predict the highs and lows of O_3_ and PM_2.5_ concentrations compared with earlier CMAQ models and spatial interpolation methods (e.g., kriging), subsequently providing an air quality database with the temporal variability indicative of air pollutant concentrations measured at central site monitors (Berrocal et al. 2012). The benefits of the increased air quality database available through the use of the downscaler CMAQ model are limited by potential exposure misclassification as a result of less confidence in the bias-corrected CMAQ predictions in rural areas because of the increasing distance of these locations from air quality monitors. This is reflected by the distribution of O_3_ monitors across the state of North Carolina during the course of the study duration. Of the 42 air quality monitors reporting O_3_ concentrations in 2006 and 2007, and 40 in 2008, approximately 69% were in Metro-Urban, 9% in Non-Metro Urban, 7% in Less Urbanized, and 14% in Rural counties (U.S. EPA 2013c). Additional exposure misclassification could be attributed to the extrapolation of predictions from the census-tract level to the county level. It is important to note that each prediction from the CMAQ model using the downscaler approach is associated with some model uncertainty. In this analysis, the uncertainty associated with each prediction was not incorporated into the statistical analysis conducted.

The present study is also potentially limited by the reporting of asthma ED visits to NC DETECT during the study duration. As previously mentioned, not all hospitals were reporting to NC DETECT in 2006, with 56.7% of the 113 EDs covered by the mandate reporting at the start of the year and 77.9% by the end of the year, with total coverage increasing to 92.5% and 99% of all ED visits reported in 2007 and 2008, respectively. The smaller number of EDs reporting to NC DETECT in portions of 2006 may not accurately reflect the statewide day-to-day changes in asthma ED visits. An additional limitation of the NC DETECT data set used in this analysis is that it did not distinguish whether the hospital visit for an individual occurred in a county far from their county of residence, which could result in exposure misclassification.

An additional potential limitation of this analysis is the modified RUCC classification scheme used to designate urban and Rural counties. It is possible that a truly Rural county is geographically adjacent to a more urban county, or a county that contains a large, metropolitan city. For example, the Northeast corner of North Carolina is very rural and lightly populated; however, Currituck County is classified as Metro Urban, and Dare County as Non-Metro Urban more than likely because of their proximity to Norfolk, Virginia. However, by using four categories of urbanicity we have attempted to minimize the concern associated with the dichotomous variables often used to designate urban and rural locations, that is, the masking of important gradations and the potential misclassification of a substantial portion of the population (Hall et al. 2006).

Although the RUCC classification scheme was used as a measure of urbanicity in this analysis, other classification schemes that use different metrics exist. For example, Urban Influence Codes capture economic opportunities of counties by taking into account county size and access to larger economies (USDA 2012a), and Rural-Urban Commuting Areas delineate sub-county components by classifying U.S. census tracts using measures of population density, urbanization, and daily commuting (USDA 2013b). Luben et al. (2009) found a high correlation among counties classified in each scheme using a four-level categorization similar to that used in the present study. Because the focus of this analysis was on examining differences in associations between O_3_ exposure and asthma ED visits by urbanization at the county level, we felt the RUCC classification scheme better represented this metric. A limitation of each classification scheme is that they were developed for national use; therefore, it is unclear how well each can draw distinctions between gradients of county-level urbanicity within a state.

## Conclusions

The present study demonstrates an alternative to using traditional air quality data to examine the association between short-term air pollution exposures and health effects, and subsequently the ability to examine a larger spatial extent. Our results are generally consistent with a growing body of evidence demonstrating an association between short-term O_3_ exposures and asthma ED visits, specifically in children. In addition, our findings suggest the association between O_3_ and asthma ED visits is not limited to large metropolitan areas and that it is stronger in counties with low health rankings, although stratified estimates had larger uncertainty with point estimates that tended to be near or below the null.
